# A Clinician-Controlled Just-in-time Adaptive Intervention System (CBT+) Designed to Promote Acquisition and Utilization of Cognitive Behavioral Therapy Skills in Bulimia Nervosa: Development and Preliminary Evaluation Study

**DOI:** 10.2196/18261

**Published:** 2021-05-31

**Authors:** Adrienne Juarascio, Paakhi Srivastava, Emily Presseller, Kelsey Clark, Stephanie Manasse, Evan Forman

**Affiliations:** 1 Center for Weight, Eating and Lifestyle Science Drexel University Philadelphia, PA United States

**Keywords:** eating disorders, telemedicine, mobile phone, smartphone, technology, cognitive behavioral therapy

## Abstract

**Background:**

Cognitive behavioral therapy (CBT) for bulimia nervosa (BN) is most effective when patients demonstrate adequate skill utilization (ie, the frequency with which a patient practices or uses therapeutic skills) and skill acquisition (ie, the ability to successfully perform a skill learned in treatment). However, rates of utilization and acquisition of key treatment skills (eg, regular eating, urge management skills, and mood management skills) by the end of the treatment are frequently low; as a result, outcomes from CBT for BN are affected. Just-in-time adaptive interventions (JITAIs) may improve skill acquisition and utilization by delivering real-time interventions during algorithm-identified opportunities for skill practice.

**Objective:**

In this manuscript, we describe a newly developed JITAI system called CBT+ that is designed to facilitate the acquisition and utilization of CBT for BN treatment skills when used as a treatment augmentation. We also present feasibility, acceptability, and preliminary outcomes data from a small proof-of-concept pilot trial (n=5 patients and n=3 clinicians) designed to identify opportunities for iterative development of CBT+ ahead of a larger ongoing randomized controlled trial.

**Methods:**

A total of 5 individuals with BN received 16 sessions of outpatient CBT for BN while using the CBT+ app. Data were collected from patients and clinicians to evaluate the feasibility (eg, app use and user adherence), acceptability (eg, qualitative patient and clinician feedback, including usefulness ratings of CBT+ on a 6-point Likert scale ranging from 1=extremely useless to 6=extremely useful), and preliminary outcomes (eg, improvements in skill utilization and acquisition and BN symptoms) of the CBT+ system.

**Results:**

Patients reported that CBT+ was a relatively low burden (eg, quick and easy-to-use self-monitoring interface), and adherence to in-app self-monitoring was high (mean entries per day 3.13, SD 1.03). JITAIs were perceived as useful by both patients (median rating 5/6) and clinicians (median rating 5/6) for encouraging the use of CBT skills. Large improvements in CBT skills and clinically significant reductions in BN symptoms were observed post treatment. Although preliminary findings indicated that the CBT+ system was acceptable to most patients and clinicians, the overall study dropout was relatively high (ie, 2/5, 40% patients), which could indicate some moderate concerns regarding feasibility.

**Conclusions:**

CBT+, the first-ever JITAI system designed to facilitate the acquisition and utilization of CBT for BN treatment skills when used as a treatment augmentation, was shown to be feasible and acceptable. The results indicate that the CBT+ system should be subjected to more rigorous evaluations with larger samples and should be considered for wider implementation if found effective. Areas for iterative improvement of the CBT+ system ahead of a randomized controlled trial are also discussed.

## Introduction

### Background

Bulimia nervosa (BN) is characterized by recurrent episodes of binge eating (ie, eating a large amount of food within a discrete period accompanied by a sense of loss of control over eating) and compensatory behaviors such as purging (eg, self-induced vomiting and misuse of a laxative or diuretic), fasting, or driven exercise [1]. The leading evidence-based treatment for BN is cognitive behavioral therapy (CBT), a present-focused, active, skill-oriented treatment. Although CBT can be an effective treatment for BN, a recent meta-analysis found that nearly 70% of patients remain at least partially symptomatic at the end of the treatment [2].

One reason many patients may fail to achieve remission from CBT for BN is suboptimal levels of therapeutic skill acquisition (ie, the ability to successfully perform a skill learned in treatment) and skill utilization (ie, the frequency with which a patient practices or uses therapeutic skills) [3,4]. Acquisition and utilization of therapeutic skills designed to reduce dietary restraint (eg, regular eating) consistently predict treatment outcomes [4-7]. In addition, numerous studies have demonstrated that reduction of dietary restraint is a key mechanism of action in CBT for BN [2,8-11]. Although less well-studied, failure to respond adaptively to cues for binge eating (particularly a failure to regulate negative affect) is also strongly associated with the maintenance of BN symptoms [12,13]. A growing body of literature has identified that the use of therapeutic skills such as mood management techniques and the resulting improvements in the ability to manage negative affect during CBT for BN are associated with symptom improvement [12,14]. Existing data thus suggest that improvements in the acquisition and utilization of skills related to reducing dietary restraint and increasing adaptive responses to cues could strongly improve treatment outcomes for BN.

Mobile health (mHealth) technologies, specifically just-in-time adaptive interventions (JITAIs), are a promising intervention paradigm that may be able to increase skill acquisition and utilization when used in conjunction with in-person treatment. JITAIs monitor the temporal dynamics of an individual’s state in real time and quickly adapt to the individual’s current contextual state to provide individually tailored interventions at crucial times of need [15]. JITAIs are increasingly being developed and used to augment treatment for several health care concerns [16-21] and generally show promise in the treatment of numerous physical [22] and mental health concerns [23]. Despite early successes in other fields, JITAIs are still relatively rare, and few have been tested as an augmentation to a comprehensive therapy package. In addition, although JITAIs are often described as providing tailored interventions, the tailoring of intervention content is typically limited to a series of prewritten interventions that are then matched to the user’s current contextual state (eg, providing a prewritten reminder to get up and walk when an app has identified that the user has not walked in a certain period). The ability of JITAIs to (1) augment a comprehensive therapy package such as CBT for BN and (2) allow for more sophisticated tailoring of abilities by enabling a treating clinician to flexibly adjust the timing, content, and goals of a JITAI to match an individual patient remains largely untested.

### This Study

As part of an ongoing National Institute of Mental Health (NIMH)–funded clinical trial (R34MH116021), our team recently developed CBT+, a JITAI system comprising a patient smartphone app and clinician portal designed to augment traditional in-person CBT for BN. In the development and design of the CBT+ system section below, we describe the core features of the CBT+ app with a particular focus on how we designed CBT+ to work as an augmentation to an ongoing treatment program by allowing the treating clinician to flexibly adjust the timing, content, and goals of CBT+ to match the changing needs of an individual patient. We also present data on feasibility (eg, app use and user adherence), acceptability (eg, qualitative patient and clinician feedback), and preliminary clinical outcomes from our initial wave of pilot patients (n=5) and therapists (n=3).

### Development and Design of the CBT+ System

#### Identifying the Optimal Ways to Extend CBT for BN

As described above, CBT for BN strives to help patients acquire and use skills designed to (1) reduce dietary restraint and (2) increase adaptive responses to cues [3]. To reduce dietary restraint, CBT for BN encourages the development of 3 core skills: (1) scheduling eating episodes at regular intervals throughout the day, with the goal of eating 3 meals and 1-2 snacks per day; (2) eating a sufficient number of calories at each meal or snack to prevent acute hunger; and (3) eating a sufficient range of food, including foods the patient may fear eating (eg, desserts and carbohydrates), to reduce or prevent feelings of deprivation. The ultimate goal of these 3 skills is to reduce several types of dietary restraint (eg, caloric restriction, hedonic restriction, and adherence to rigid food rules) that increase vulnerability to binge-eating episodes. To increase adaptive responses to cues, CBT for BN also teaches 3 core skills: (1) how to recognize internal (eg, negative affect and urges) and external triggers (eg, presence of palatable foods) for BN symptoms, (2) how to modulate mood when experiencing negative emotions (without resorting to binge eating), and (3) how to manage urges to binge or use a compensatory behavior. These 3 skills are designed to help patients identify internal and external triggers for binge eating before these triggers lead to a binge-eating episode and successfully use therapeutic skills to manage negative affect and urges [3].

We developed the CBT+ JITAI system as a CBT for BN treatment augmentation with the goal of increasing the utilization and acquisition of the 6 core skills described above. Accordingly, the full CBT+ JITAI system consists of 2 highly integrated subsystems: (1) the CBT+ patient smartphone app (CBT+ app) and (2) a CBT+ website that is accessible by the CBT therapist (CBT+ clinician portal). Below, we describe both the subsystems in more detail.

#### CBT+ App

##### CBT+ Electronic Self-Monitoring Forms

**Figure 1:**
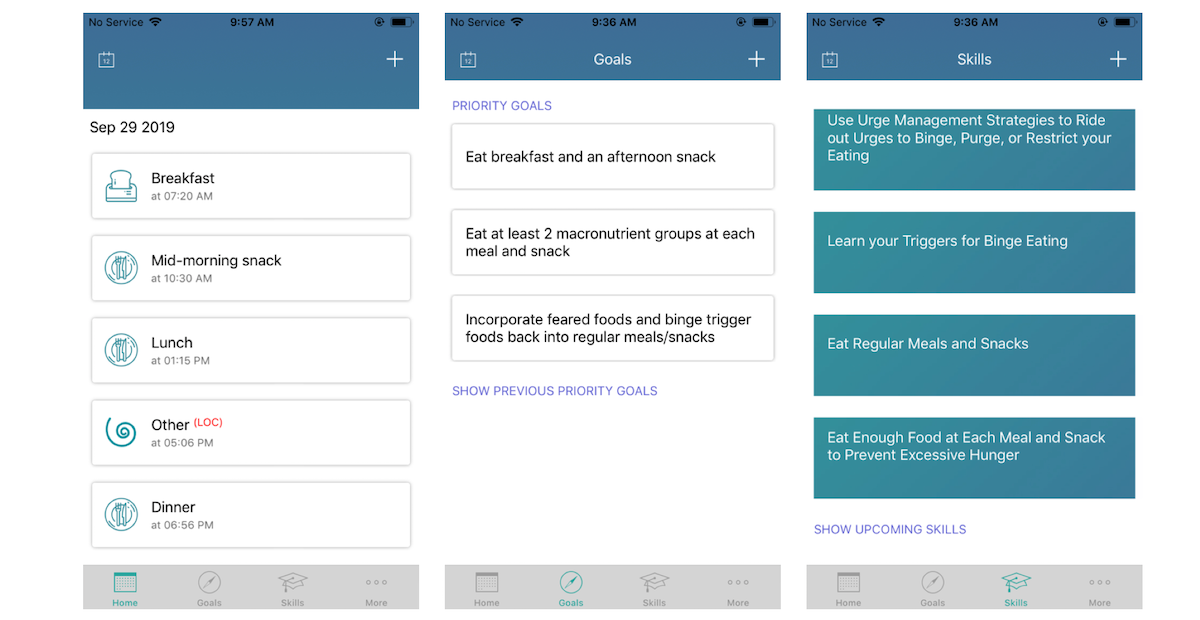
Screenshots of the home screen showing all the tabs CBT+ users can access.

**Figure 2:**
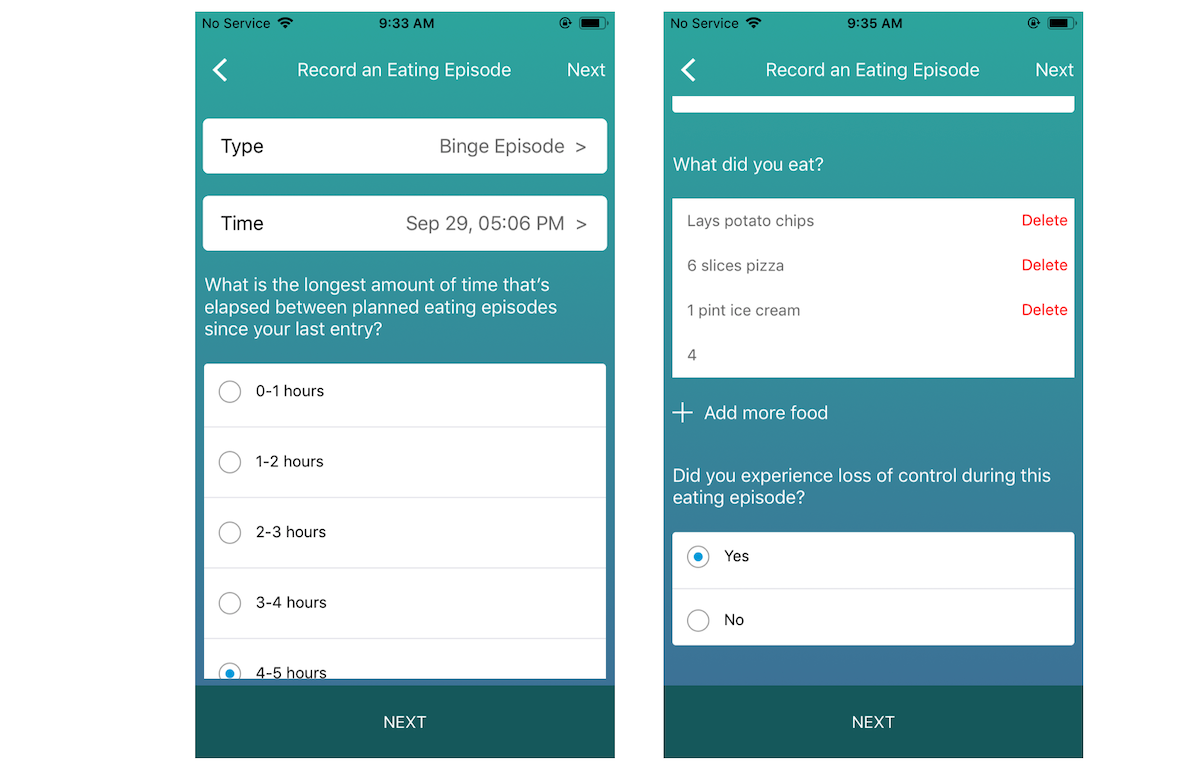
Screenshots of the electronic self-monitoring form of the CBT+ smartphone app.

The CBT+ app (Figure 1) was designed to replace the traditional paper-based monitoring forms used in CBT for BN with easy-to-use electronic self-monitoring forms (Figure 2). Individuals were instructed to record all of their eating episodes in real time to capture information about their eating pattern, including the type of meal (eg, breakfast, midmorning snack, and lunch), time of the meal and snack, and food consumed. At each meal entry, patients also reported the presence or absence of any disordered eating behaviors or urges and rated their current mood. Individuals were also instructed to complete an *other* entry any time they engaged in disordered eating behavior outside of meal entries (eg, laxative use and excessive exercise), experienced a strong urge to engage in a disordered eating behavior, or experienced a significant change in mood. An open text box is available at all entries for patients to add additional context and comments if desired. The data entered into the electronic monitoring form provide the momentary data needed to power the clinician-controlled JITAI algorithm (described in greater detail in *JITAI Algorithm* section below).

##### Goals

The goals feature allows patients to view up to 3 weekly goals (called priority goals in the app) that they collaboratively set with their clinician during treatment sessions. Priority goals are a method by which the clinician can control the JITAI algorithm, as individuals will only receive momentary interventions that have been linked with a priority goal. Priority goals are typically (though not always) linked to 1 of the 6 CBT skills described above, but clinicians can scale up or scale down the goal to meet the needs of an individual patient. For example, although regular eating skills recommend patients eat 3 meals and 1-2 snacks per day, the priority goals for regular eating can be more modest at first (eg, eating 2 meals and 1 snack) for patients who need to slowly build up to 3 meals and 1-2 snacks per day over several weeks. Weekly goals can be viewed on the goals tab of the CBT+ app. Before attending each therapy session, patients were instructed to rate how successful they were in meeting their goals each week, which provides information to the treating clinician that can be used to inform goal progression.

##### JITAI Algorithm

Each time an individual enters data, an embedded algorithm determines whether there is an opportunity for the individual to practice a therapeutic skill. The starting algorithm is based on CBT theory [24] and triggers interventions when self-monitoring logs suggest that CBT skills practice would be beneficial. For example, if one of an individual’s priority goals is to *practice urge management skills whenever you experience an urge to binge*, the CBT+ algorithm will check self-monitoring records at each entry and suggest an urge management skill to try whenever an urge is reported. As described above, the starting algorithm can be flexibly adjusted by the clinician by altering the priority goals set for that week. Clinicians can also control how often the algorithm intervenes directly by scaling up or down the frequency of interventions. For example, the starting algorithm prompts users to regularly eat if 5 or more waking hours have passed without an eating episode. However, if a clinician determines that a specific patient would benefit from a more frequent reminder, they can adjust the algorithm timing. By allowing clinicians to flexibly adjust the embedded algorithm, CBT+ will only intervene when opportunities are identified to practice the specific skills that the clinician and patient are working on together in the therapy sessions. For example, in the earlier scenario where an urge management skill was recommended, the patient may have also reported feeling sad. However, because the clinician and patient have not yet begun working on mood management skills and no priority goal was set in this domain, the algorithm will not intervene on this practice opportunity.

##### JITAI Interventions

**Figure 3:**
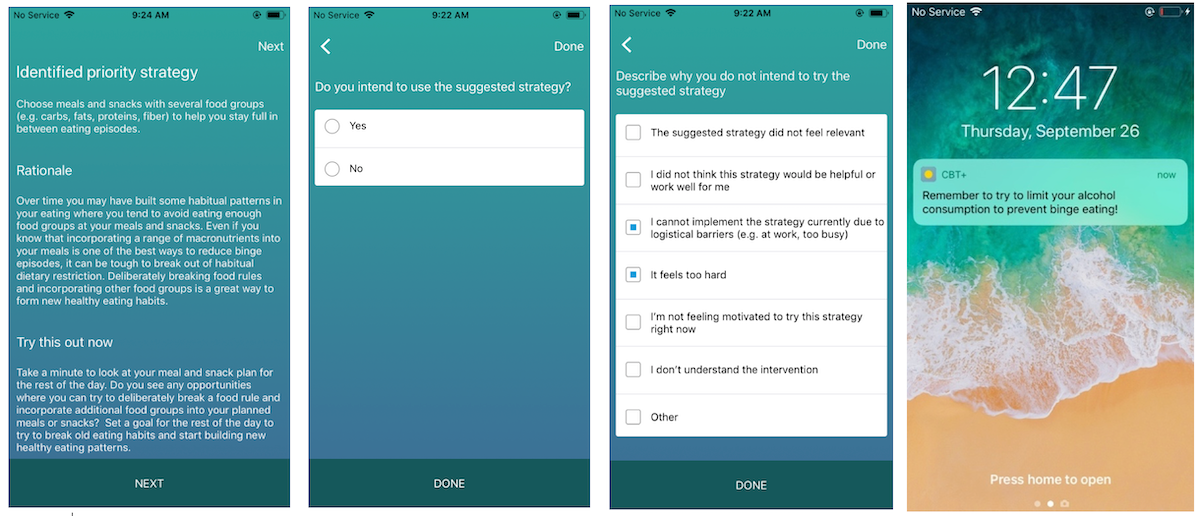
Screenshots of the delivered just-in-time adaptive interventions and follow-up questions.

All CBT+ interventions follow a specific structure that is designed to facilitate awareness of when, why, and how to use a specific skill at the moment that the skill is suggested. Each intervention has 3 key components: (1) the skill identified as most important to practice based on the data entered by the patient; (2) a brief rationale explaining why the patient would benefit from practicing that skill in the current moment; and (3) instructions to *try this out now,* which provide guidance on how to implement the suggested skill at the current time. Additional information about how clinicians can control the specific content of the interventions will be discussed in the *Clinician Portal* section below. Every time an intervention is delivered, patients are asked if they intend to use the recommended skill. If patients report that they do not intend to try out the recommended skill, they can select a reason (eg, *the suggested strategy did not feel relevant* and *it feels too hard*) that will be shared with their treating clinician and can be used to refine future intervention content. In addition to the algorithm-generated interventions, patients can also receive push notification interventions at scheduled times preset by their treating clinician. Push notification interventions will be discussed further in the *Clinician Portal* section below. A standard intervention example and a push notification example are shown in Figure 3, and 2 examples of patient self-monitoring log entries and a corresponding intervention can be found in Textbox 1.

**Examples of events that would trigger just-in-time adaptive interventions (JITAIs) and examples of corresponding JITAIs.**

- Patient X had a priority goal of *eat regular meals and snacks* on her self-monitoring form. She noted that it had been more than 5 hours since her last meal or snack. The algorithm identified this as an opportunity to reinforce using regular eating skills and delivered a just-in-time adaptive intervention (JITAI; for example, to address logistics or problem-solving issues using regular eating skills).
  On the basis of your most recent data, CBT+ has identified a strategy you can try out now to build your skills:
  Identified priority strategy: Stick to your planned meal and snack schedule, even if you are worried it is too much food or that you may gain weight.
  Rationale: Getting back on track with your regular eating plan after you missed a meal or snack because of fears of weight gain often requires some problem solving.
  Try this out now: Take a minute to think through how you will meet your regular eating goals for the rest of the day. Are there any logistical barriers you may experience? If so, try to identify some options for how you may be able to work around some of these barriers so that you can meet your goals.
- Patient Y had a priority goal of *learning to manage negative emotions skill*, and on her self-monitoring form, she gave a rating of 4 on the mood rating question (mood range 1 [good]-5 [bad]) and also checked off *no* box in response to the question “have you used a mood management strategy?” The CBT+ algorithm identified this as an opportunity to practice a mood management strategy and delivered a JITAI (eg, encouragement for using alternative activities strategies for mood management).
  On the basis of your most recent data, CBT+ has identified a strategy you can try out now to build your skills:
  - Identified priority strategy: Engage in alternative activities to improve your mood.
  - Rationale: Breaking out of certain problematic habits (eg, engaging in an alternative activity instead of binge eating when feeling sad) requires tolerating uncomfortable thoughts, urges, and/or cravings. Learning to tolerate distress is key to long-term success.
  - Try this out now: Many people find reflecting on their long-term goals to be helpful in tolerating distress at the moment. How will engaging in an alternative activity right now make your life better in the long term, even if it feels uncomfortable in the short term? Remember that thoughts, feelings, urges, and cravings cannot physically hurt you, and engaging in an alternative activity will take you closer to reaching your long-term goals. Practice tolerating distress and engaging in an alternative activity from our list right now.

##### On-Demand CBT Skills

The CBT+ app also includes a repository of the skills that individuals have learned in therapy to date. Individuals can always access a brief description of what each skill is and how to use that skill.

#### CBT+ Clinician Portal

##### Patient Dashboard

**Figure 4:**
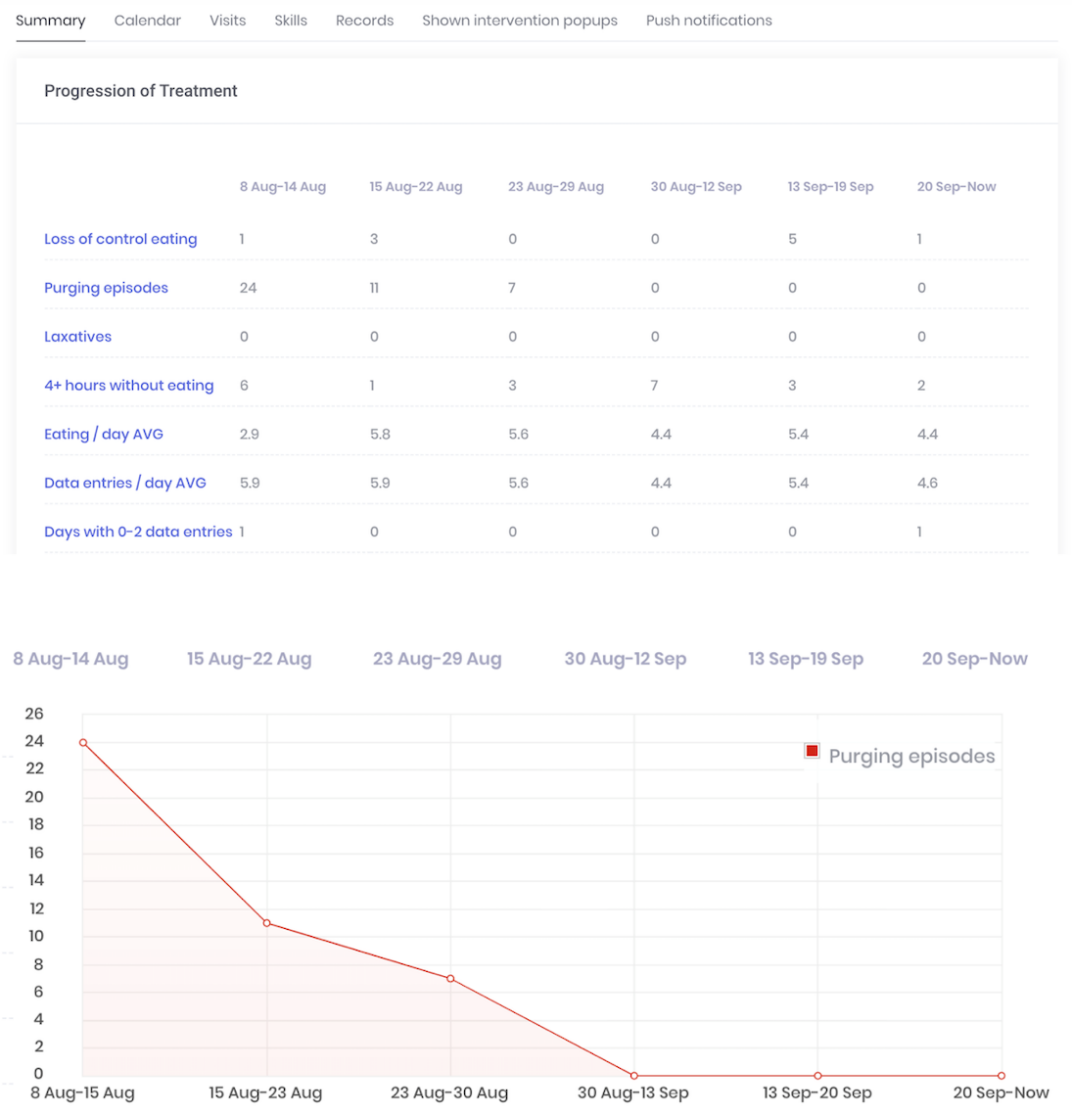
Patient dashboard of the CBT+ clinicians’ portal.

The patient dashboard quickly summarizes key data from the patients last week (eg, number of binge episodes and number of self-monitoring entries). Clinicians can also view trends in their patients’ disordered eating behaviors over time through auto-generated graphs by clicking on any dashboard summary. A screenshot of the patient dashboard and a trend graph are shown in Figure 4.

##### Self-Monitoring Records

**Figure 5:**
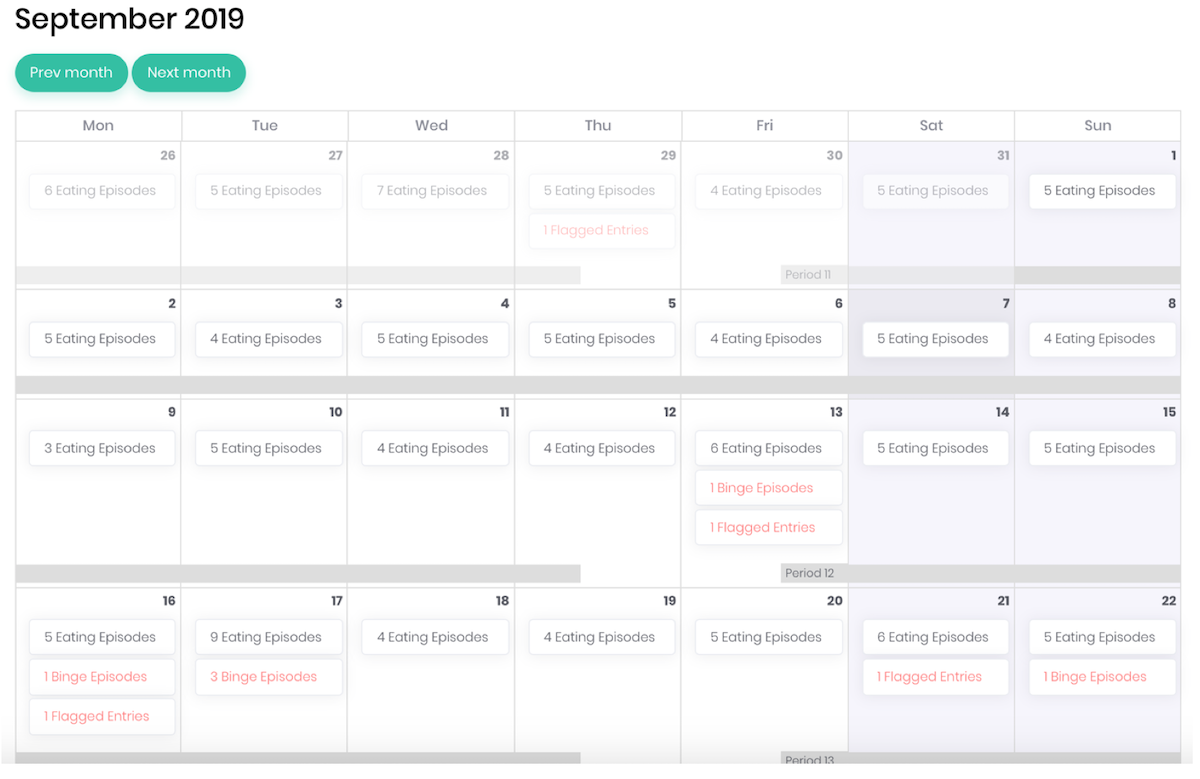
Calendar feature of the CBT+ clinicians’ portal.

Clinicians can use a calendar feature in which daily self-monitoring records can be viewed. All eating disorder behaviors endorsed by the patient are flagged in red to allow clinicians to quickly view the data from the past week and identify which days are most important to discuss during the therapy sessions. Figure 5 shows an example of the calendar feature.

##### Skills Tab

Clinicians can use the skills tab to open new skills once they have been introduced into therapy. Once a skill has been introduced during a session, the skill becomes viewable in the CBT+ app skill section, and priority goals that intervene in this skill can be assigned.

##### Interventions Tab

In the interventions tab, the clinician can view which interventions their patients received in the past week. Clinicians can also view if their patients intend to implement the suggested strategy and the reasons for not implementing a recommended strategy. This tab can be used to understand which intervention the patient found helpful or provide guidance for adjusting intervention content over time to address the reported barriers to skill use.

##### Push Notification Intervention Tab

The push notifications tab on the patient dashboard shows a list of all push notifications that have been sent or are scheduled to be sent to the patient. There are 2 types of push notifications: self-monitoring push notifications that prompt patients to enter data if they have not done so for a set number of waking hours (default is set at 5 hours, but this is adjustable by clinicians) and custom push notifications. Custom push notifications allow clinicians to set a one-time or recurring reminder to practice a therapeutic skill. These notifications allow clinicians to preset JITAIs that are not dependent on self-monitoring log completion. Custom push notifications can be used to remind patients ahead of sessions to try out a specific homework at agreed-upon times, encourage skill use during known high-risk times (eg, right before a scheduled weekly happy hour and on weekday evenings where unscheduled time can be a common trigger for binge-eating episodes), or send motivational messages.

##### Visits Tab

Clinicians use the visits tab to see the summary of goals that have been assigned to the patient at each session and record session notes. During each in-person therapy session, clinicians add a new visit and set priority goals for the period between the current visit and the next therapy session. As described above, priority goals are typically linked to 1 of 6 CBT skills. Linking to 1 of the 6 skills allows clinicians to view a list of prewritten interventions that can be delivered during algorithm-identified moments when that skill should be practiced. The prewritten interventions contain suggestions for specific CBT strategies that may facilitate skill use, and they are designed to address common barriers to skill implementation (ie, low accountability, poor awareness of when and how to use skills, habitual use of maladaptive coping skills, distress intolerance, poor problem-solving abilities, low motivation, and poor memory). Clinicians can choose any or all of the prewritten interventions that may be relevant for their patient, or if none of the prewritten options are relevant, they can create their own custom intervention. After assigning weekly goals and linking goals to interventions, clinicians save and close the visit, which triggers an automatic update to the individual patient’s CBT+ algorithm.

## Methods

### Study Design

We conducted a small pilot trial (n=5 patients and n=3 clinicians) to assess the feasibility and acceptability of augmenting CBT for BN with the CBT+ JITAI system and identify opportunities for improvement to CBT+ ahead of a larger ongoing randomized controlled trial (RCT). In this study, we present data obtained from clinicians and patients that were used to inform iterative development.

### Participants

Patients were eligible if they were aged between 18 and 70 years and met the criteria for a primary diagnosis of BN. Exclusion criteria included active severe psychiatric comorbidity that would limit the ability to participate in an outpatient clinical trial for BN (eg, psychosis, acute suicidality, and severe substance use disorder), inability to speak and write English, diagnosis of an intellectual disability that would impair the use of the CBT+ app, or having received a full trial of CBT for BN in the past. Although the CBT+ app is currently only compatible with Apple iPhone iOS, participants were not excluded based on ownership of an iPhone; participants without iPhones were given loaner iPhones to use for the duration of the study. Patients’ (n=5, 4 females and 1 male) average age was 35.6 (SD 6.8, range 25-42) years and average BMI was 31.2 (SD 4.43, range 25.8-37.1) kg/m^2^. A total of 4 patients self-reported as Caucasian or White, 1 identified as multiracial, and 2 identified as Latino or Hispanic.

Clinicians were either masters (n=2) or doctoral-level clinicians (n=1) with a minimum of 2 years of experience in delivering CBT for BN. All 3 clinicians previously completed the Centre for Research on Eating Disorders at Oxford web-based training program developed by Fairburn et al [25] and received weekly supervision from a licensed psychologist during the course of the treatment trial.

### Treatment

The treatment consisted of 16 sessions of CBT for eating disorders based on the treatment approach developed by Fairburn [24]. In addition to in-person treatment, all patients were instructed to use the CBT+ app daily for the duration of the clinical trial.

### Assessments

**Figure 6:**
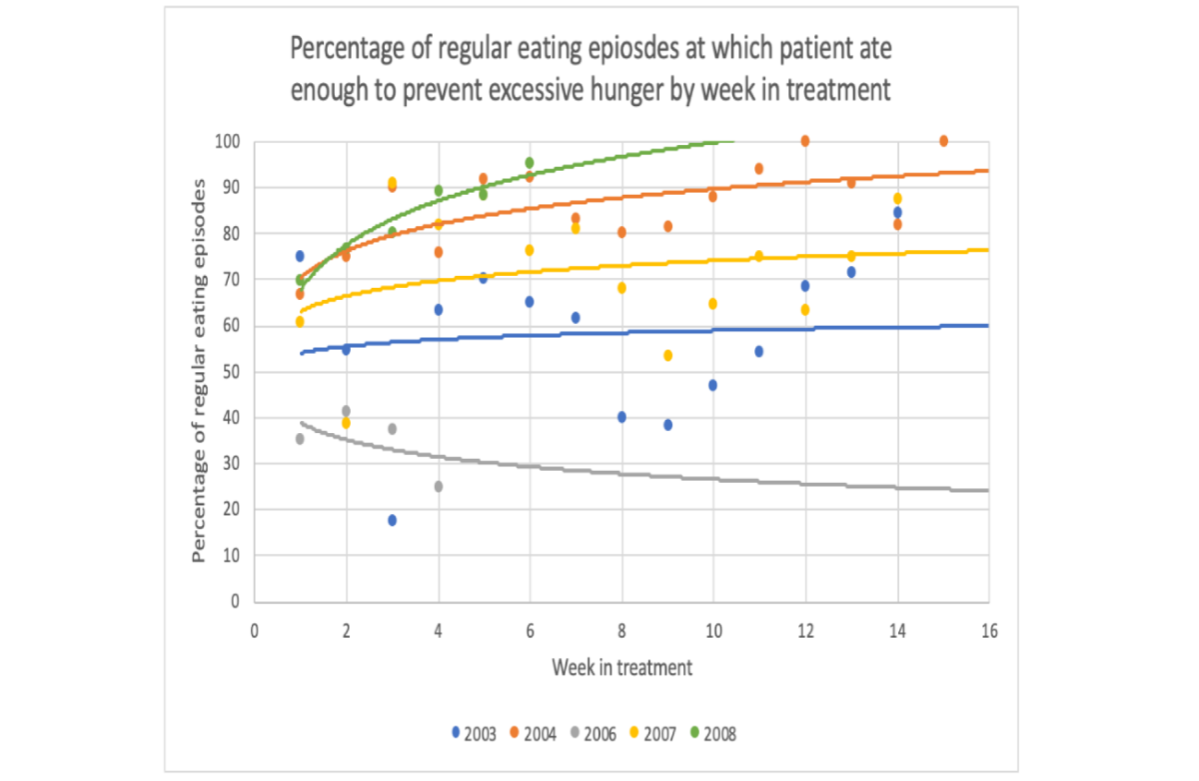
Change in frequency with which patients endorsed eating enough at regular eating episodes throughout treatment.

**Figure 7:**
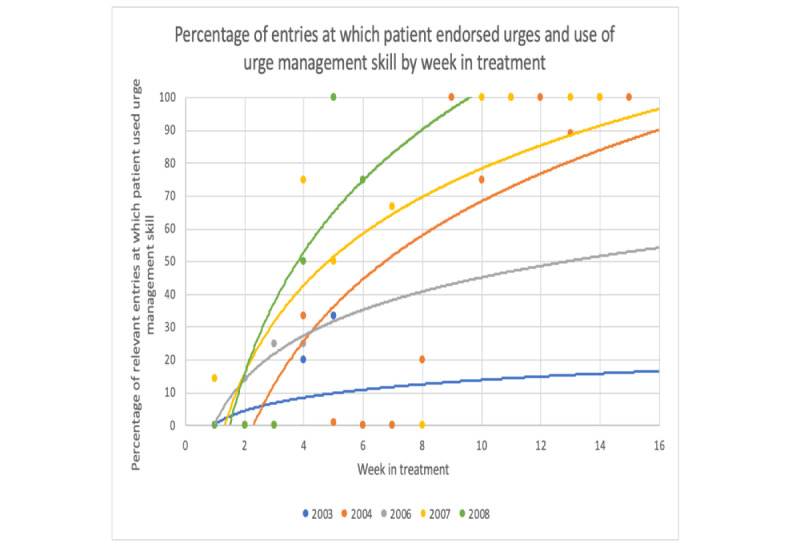
Change in frequency of reported urge management skill use throughout treatment.

Full assessments occurred at baseline, midtreatment (week 8), and posttreatment (week 16). Assessments included the Eating Disorder Examination (EDE, version 17.0) [26]; a series of self-report measures, including the Quality of Life Inventory (QOLI) [27]; and behavioral tasks. Patients completed phone interviews after 4, 8, 12, and 16 weeks of treatment to provide specific feedback on the CBT+ app. Patients also completed weekly presession questionnaires in which they rated different components of the CBT+ app, reported on their skill use in the past week, and provided qualitative feedback about the app. To measure dietary restraint and emotion regulation, patients completed the EDE questionnaire restraint subscale items [28] and 4 individual items selected from the Difficulties in Emotion Regulation Scale [29] in the weekly presession questionnaires. Clinicians completed postsession questionnaires after every session and rated the usefulness of the CBT+ system. To quantify CBT+ app use, the number of days the app was used (ie, the number of days when at least one entry was completed) and the number of entries completed per day (ie, adherence) were calculated. For this aim, we only included days during which the patient was still enrolled in the trial to allow for the determination of app use during the period when the patient intended to use the CBT+ system. Thus, for the 2 patients who discontinued treatment, we only included use data up to the date that they discontinued participation in the study. Data from the app were used to examine skill use during each treatment period. To examine the use of the skill *eating enough at each meal and snack to prevent excessive hunger* throughout the treatment, we calculated the weekly percentage of regular eating episodes (ie, excluding those accompanied by loss-of-control over eating) where participant endorsed that they “ate enough food to prevent excessive hunger before [their] next planned eating episode” and/or that they “ate a range of macronutrients (eg, protein, fat, carbs)” (Figure 6). The percentage of records in which the participant endorsed using an urge management skill when relevant (ie, when the participant also experienced an urge to binge or use a compensatory behavior) was calculated for each week in treatment to identify changes in the use of urge management skills throughout treatment (Figure 7).

## Results

### Study Feasibility

Of the 7 patients who were eligible to participate in the study after the baseline assessment, 5 chose to enroll in the study. Two patients who began treatment dropped out of the study: one after session 4 and the other after session 7. Both stated that their reason for dropping out was being too busy to continue treatment. The remaining patients (n=3) completed all 16 treatment sessions and all assessments.

### Feasibility of the CBT+ System

In weekly presession questionnaires, patients reported a few minor technical issues with the CBT+ app (eg, occasional app crashing and occasional occurrences of an entry not saving), but no other major problems with the app were reported. Patients also noted occasional minor issues using the CBT+ system that were not because of technical issues with the system itself but still prevented app use (eg, forgetting login information and poor Wi-Fi connectivity). Technical issues that limited the use of the CBT+ app for at least one entry were reported in 5% (3/58) of weekly patient questionnaires. Clinicians reported only 1 bug with the clinician portal: eating episodes were occasionally mistakenly flagged as binge episodes on the calendar page. This bug was reported in 3% (2/58) of the weekly clinician questionnaires. Given that the technical issues with the CBT+ system were minor and did not interfere with treatment delivery, no live changes in the system were made during this study. As described above, CBT+ app use was evaluated during the periods when a patient was active in the study (eg, for the 2 users who discontinued in-person treatment, data on their app use were included until the time they discontinued study participation). Although we considered reporting adherence across 16 weeks for all participants, regardless of whether they completed treatment or not, we ultimately decided that understanding app adherence during active treatment would be more valuable for future iterations than reporting app adherence overall, which would mix the results of those who intentionally used the app with those who intentionally discontinued participation and thus limit interpretability. On average, patients used the app on 86.1% of days (range 74.6%-92.9%; SD 7.5%) and made 3.13 entries per day (range 2.53-4.34; SD 0.74) while they were actively in treatment. User adherence declined somewhat throughout the course of the treatment but remained high among patients who continued their study participation. All 5 patients used the app on 100% of days and completed 3.59 entries per day (range 2.67-4.88; SD 0.82) during the first week of treatment. By week 8 (n=3), this number had declined to 89.4% of days (range 68%-100%; SD 18.4%) and 2.77 entries per day (range 2.20-3.88; SD 0.96). By the final week of app use, patients (n=3) used the app on 85.5% of days (range 78%-90%; SD 6.78%) and made an average of 3.19 entries per day (range 2.80-3.44; SD 0.34).

### Acceptability of the Overall CBT+ System

Data from all patients were included in acceptability analyses and included the final acceptability interviews completed with the 2 patients who discontinued treatment. Patients’ median rating of the overall usefulness of CBT+ was 5 (out of 6; IQR 1) on a 6-point Likert scale ranging from 1=extremely useless to 6=extremely useful. During qualitative feedback interviews, all patients reported that the app was easy to use and that they liked that the app kept them on track with their weekly goals and reinforced the use of strategies. Textbox 2 shows additional qualitative feedback from patients. Key themes that emerged from qualitative interviews with patients included positive feedback regarding the ability of CBT+ to provide added accountability and awareness of the patient’s own patterns, ability to track progress, and reinforcement of skills and strategies. Negative feedback primarily centered on the redundancy of interventions and concerns regarding the burden associated with completing daily records. Patients reported that self-monitoring via the CBT+ app could be burdensome, partially because tracking food intake is in itself burdensome, regardless of the format used, but also partially because of the number of questions asked in the CBT+ app at each entry. Patients also reported that they were less likely to thoroughly read the CBT+ intervention texts as treatment progressed because they had already read a similar version of the intervention before and found that they did not need to reread the intervention content.

**Qualitative feedback from patients on the CBT+ app.**

**Accountability and Identifying Patterns**

- “Tracking my food intake allows me to check in with myself throughout the day.”
- “I like being prompted to reflect on how I’m feeling.”
- “It’s a good record to look back on and see what was happening if I fall off with my goals.”
- “It helps us to have more honest discussions and to validate what [I] think with actual data.”
- “It reminds you and when you reflect back...it helps to put things into perspective and see why things are happening.”
- “The fact that the app says, ‘Did this include two...or three [food] groups?’ helps you to pay attention to what you’re eating.”
- “It was super helpful. It made me think about [eating enough] more and made me be more cognizant of what I was putting in my meals.”
- “I could start to see patterns by recording everything.”
- “It helps you to understand why you’re eating so much in a certain moment. I always use the box at the end to make notes about whether something is happening and where I am. Writing it down and reflecting on it helps.”
- “It is helpful since I have to fill out the form and I have to reflect on everything that happened when I ate the meal. It definitely helps me realize why I ate what I did or why I ate as much as I did. It helps me realize why I was so hungry, if I didn’t eat for the last 5 hours.”

**Tracking Progress**

- “I like that it provides structure and feedback on my progress. It keeps me on track with my goals.”
- “[The app] helped by reminding me to record and being able to see my progress prompted me to maintain that progress.”

**Reinforcement of Skills and Strategies**

- “I like how the app reinforces the use of strategies.”
- “I think it helps to keep you aware and reflective about what you discuss in your therapy sessions. Like it keeps it in the back of your mind. What you’re eating and putting into the app is very purposeful and connected to what you discuss in therapy.”
- “I find [the identified priority strategies] somewhat helpful in changing my behavior.”
- “[The app] helps because it reminds you of [urge management strategies].”
- “I’m not the best at making entries in the moment, I think [the app] would be more useful if I actually did that. Otherwise, it’s less useful for me if I make an entry after. But overall, having it there and having to make the entry later certainly makes you more mindful [of strategies].”
- “I’d say the app and the therapy are just as helpful as each other with [incorporating feared foods]. Because again it keeps it in the back of my mind when I have to enter the stuff in.”

**Redundancy and Burden**

- “Over the last four weeks [of treatment] it was kind of redundant and burdensome because it was the same stuff that I had been doing...It just felt kind of taxing to do it.”
- “It was a little cumbersome to record everything I ate.”
- “It’s just really burdensome, the same questions over and over again. I almost wish it was all on one page, because I feel like [clicking] next, next, next just makes it feel longer.”
- “A lot of times the same strategies kept coming up so I started ignoring them after a while.”
- “I find [the identified priority strategies] helpful but also a little repetitive. But at the same time, you want to be reminding of the strategies.”
- “I liked that at the beginning. I feel like later on [in treatment], I wouldn’t read them because I had read them a bunch of times and I’d seen them and I knew what they were talking about.”
- “I definitely find [the reminder push notifications] helpful, but a lot of times the push notifications just reinforce to me how burdensome it is. It’s almost overwhelming. It’s just another thing on my to-do list.”

Clinicians’ median rating of the overall usefulness of CBT+ was 5 (out of 6; IQR 0), using the same Likert scale described above. Clinician interviews were overall positive, but a few suggestions for improvements to the clinician portal were noted, including changing the layout for viewing self-monitoring data and flagging compensatory behaviors on the calendar page in addition to binge episodes.

### Acceptability of JITAI

The frequency of JITAIs was evaluated during the periods when a patient was active in the study (eg, for the 2 users who discontinued in-person treatment, data on JITAI frequency were included until the time they discontinued study participation). Patients received an average of 2.14 push notification reminders to enter data (range 1.76-2.69, SD 0.47) and 0.78 interventions (range 0.41-1.70, SD 0.53) per day. Table 1 shows the average percentage of total interventions associated with each skill. Significantly more interventions were received for the 3 skills related to reducing dietary restraint (1461/1590, 91.89% of all interventions) than for the 3 skills related to increasing adaptive response to cues (65/1590, 4.09% of all interventions). This imbalance was primarily driven by regular eating interventions (1288/190, 81.01% of all interventions), which may be partially due to the fact that it is (1) the first of the 6 skills introduced in CBT for BN and (2) considered an essential skill to prioritize in CBT for BN; thus, many clinicians maintained regular eating as one of the key priority goals for the majority of treatment. A secondary factor that may explain the imbalance is that 2 of the 5 patients in the pilot trial dropped out of the treatment before they were introduced to several adaptive responses to cues skills, which were introduced in the latter half of the treatment.

**Table 1:** The average percentage of total interventions associated with each skill.

Cognitive behavioral therapy skills	Interventions (%)		
	Median	25th percentile	75th percentile
Eat regular meals and snacks	86.0	57.0	96.3
Eat enough food at each meal and snack	9.5	0.2	24.4
Incorporate feared foods and binge-trigger foods	0.0	0.0	1.4
Use urge management strategies to cope with urges	3.3	0.9	5.9
Learn your triggers for binge eating	0.0	0.0	0.7
Learn to manage negative emotions	0.0	0.0	1.5

Clinicians created custom push notifications during 16% (9/56; range 0/4, 0%-5/6, 83%) of sessions. The most common type of custom push notification was a personalized reminder notification to complete self-monitoring entries as close to their eating episodes as possible or to make an *other* entry as soon as they engaged in any disordered eating behavior. The second and third most common custom push notifications were reminders to adhere to treatment recommendations (eg, “refrain from weighing yourself at home”) and interventions designed to address common treatment-interfering behaviors (eg, “try to avoid drinking too much alcohol on the weekend to prevent binge eating”), respectively.

Overall, patients reported that they intended to use the suggested strategies 97.1% (372/383) of the time when identified priority strategies were delivered (range 84/88, 95.5%-13/13 and 91/91, 100%). During qualitative interviews, 100% (5/5) of patients reported that the JITAIs provided situationally relevant interventions, although 40% (2/5) of patients described the interventions as *repetitive* and reported that they frequently received interventions related to the same types of problematic behaviors.

Descriptive statistics were calculated for the patients’ and clinicians’ ratings of the app’s usefulness. Patients’ ratings of the usefulness of JITAIs for each of the 6 skills are shown in Table 2. Patients’ median usefulness ratings for the 6 skills ranged from 4 to 5 on a 6-point Likert scale (ranging from 1=extremely useless to 6=extremely useful).

**Table 2:** Median patient ratings of the usefulness of CBT+ for the acquisition of each cognitive behavioral therapy skill.

Cognitive behavioral therapy skills	Participant rating		
	Median	25th percentile	75th percentile
Eat regular meals and snacks	5	5	5
Use urge management strategies to cope with urges	4	4	5
Eat enough food at each meal and snack	4	4	5
Learn your triggers for binge eating	4	4	5
Learn to manage negative emotions	4	4	4
Incorporate feared foods and binge-trigger foods	4	4	5

### Preliminary Clinical Outcomes

**Figure 8:**
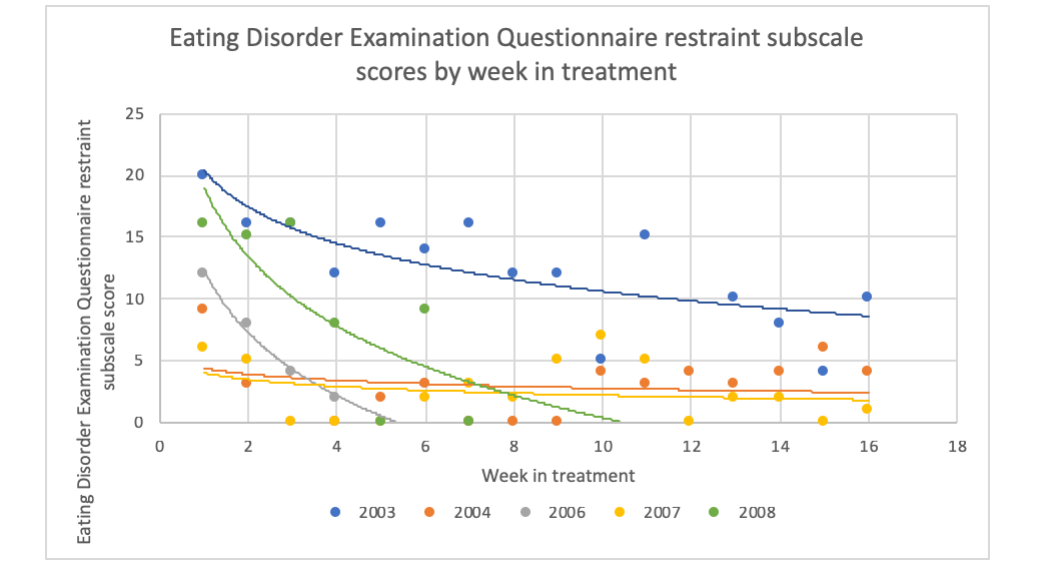
Change in weekly Eating Disorder Examination Questionnaire restraint subscale score throughout treatment.

**Figure 9:**
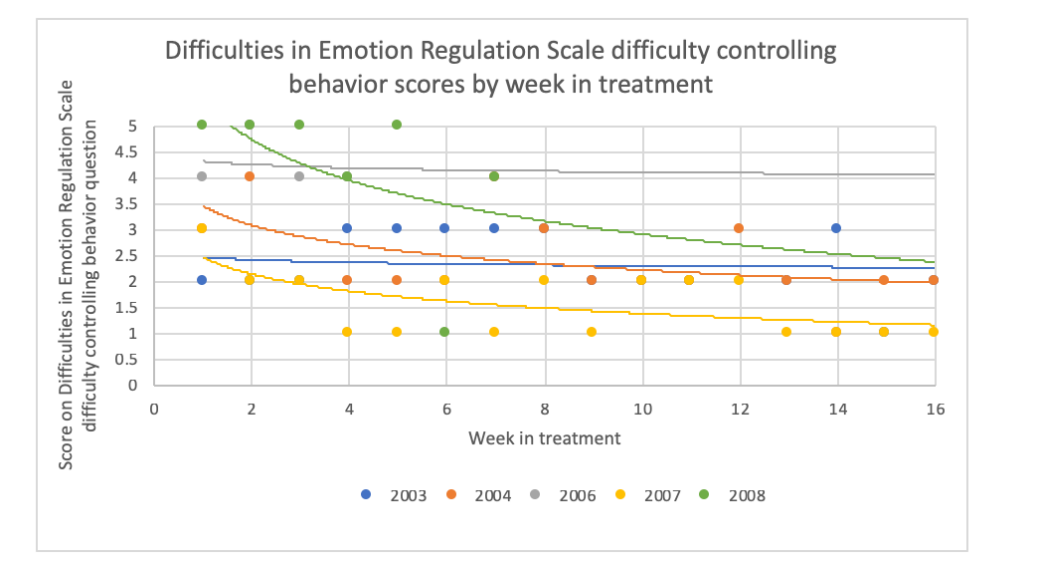
Change in score on the Difficulties in Emotion Regulation Scale item, “When I was upset, I had difficulty controlling my behaviors,” throughout treatment.

To examine changes in CBT skills during the treatment period, we graphed data collected via the CBT+ app that reflects several CBT skills. Of note, not all skills were measured weekly using the app. For these skills, we used data from the weekly self-report questionnaires described in the *Assessment* section. Figures 8 and 9 show 2 examples from the weekly questionnaires. All skills examined via app or weekly questionnaires showed a visual pattern of improvement throughout the treatment.

For clinical outcomes, we only included data from treatment completers, as both patients who dropped out discontinued their participation in the study before the midtreatment assessment point, and thus, we only had baseline EDE and QOLI scores. From pre- to posttreatment, patients who completed treatment demonstrated large decreases in the frequency of binge eating and compensatory behaviors and in EDE global scores. The detailed results are presented in Table 3. At posttreatment, the mean EDE global score for treatment completers fell within 1 SD of community norms. Although paired samples *t* tests from pre- to posttreatment did not demonstrate statistically significant changes, the effect sizes were large for eating disorder symptoms. Patients generally demonstrated slight improvements in the QOLI total score from pre- to posttreatment with a small effect size (Table 3).

**Table 3:** Change in eating disorder symptoms and quality of life from pre- to posttreatment.

Eating disorder symptoms and quality of life	Pretreatment, median (range)	Posttreatment, median (range)	*t* test (*df*)	*P* value	Cohen *d*
Past month objective binge episodes	4 (2-14)	0 (0-1)	1.65 (2)	.24	0.95
Past month total loss of control eating episodes	8 (4-14)	0 (0-1)	2.62 (2)	.12	1.52
Past month total compensatory behaviors	10 (2-10)	1 (0-4)	1.47 (2)	.28	0.85
Global Eating Disorder Examination score	2.59 (2.18-3.88)	1.73 (1.64-1.86)	2.06 (2)	.18	1.18
Quality of Life Inventory total score	15.28 (14.78-17.28)	16.75 (14.22-18.00)	−0.62 (2)	.60	0.36

## Discussion

As part of an ongoing NIMH-funded clinical trial (R34MH116021), our team developed CBT+, a JITAI system consisting of a patient smartphone app and clinician portal designed to augment traditional in-person CBT for BN. CBT+ is the first JITAI system designed as an augmentation to CBT for BN with the specific goal of promoting skills acquisition and utilization.

### Principal Findings

Our preliminary evaluation demonstrates that it is broadly feasible and acceptable to integrate a JITAI system with CBT for BN, although some concerns were noted with 2 out of 5 patients discontinuing participation in the study. Among treatment completers, outcomes were positive in all 3 patients, showing substantial decreases in objectively large binge episodes and compensatory behaviors, and average posttreatment EDE global scores were within 1 SD of community norms, a metric commonly used to define remission [30,31].

We found that user adherence to app use and data entry on the CBT+ app were high throughout the study period for patients who remained in the study. Although there was a modest fall-off in adherence rates over time, patients used the app on 87.2% (470/539) of the days, even during the last weeks of prescribed app use in treatment, which is greater than that reported in other studies (eg, the overall percentage of the days when smartphone app was used for breast cancer=45% [32] and for depression=66% [33]) and clinical reviews of mHealth apps developed to augment treatments [34]. Devising a system that facilitates continued adherence during many months is critical for JITAIs, as JITAI algorithms rely on these data to inform the timing and content of intervention delivery. High user adherence may have been because of 3 key strengths in the design of the CBT+ app. First, the user interface of CBT+ was specifically designed to be low burden, that is, patients only needed to enter a few key variables at each data entry, which allowed us to keep entry time between 1 and 2 minutes per entry. Second, the self-monitoring data were directly relevant to the in-person treatment component, and therapists routinely referred to and made use of app-collected information during treatment, which allowed therapists to reinforce frequent use of the CBT+ app. Third, patients reported finding the JITAIs helpful, which may have increased the likelihood of completing data entry because of the perceived usefulness of the JITAI system.

Qualitative feedback and weekly questionnaire results suggested that both patients and clinicians believed that the CBT+ system was easy to use and helped them keep track of their goals. With regard to the acceptability of JITAIs, CBT+ algorithms produced slightly less than 1 intervention per day. Patients perceived the JITAIs to be accurate and appropriate to the entries they completed and found the JITAIs to be helpful in promoting the use and acquisition of therapeutic skills. JITAIs designed to reduce dietary restraint comprised the majority of interventions that were delivered. This finding suggests that most patients needed continued support to practice skills designed to reduce dietary restraint (such as eating regular meals and snacks and eating enough food at each meal and snack) compared with strategies designed to reduce unhelpful responses to cues to binge eating (such as learning to manage triggers for binge eating and learning to manage negative emotions). As the reduction of dietary restraint is the most well-established mechanism of action in CBT for BN [2,8], JITAIs designed to improve skills related to reducing dietary restraint may be the most impactful.

One reason that clinicians and patients may have found CBT+ beneficial was the high customizability and personalization allowed by the JITAI system. Previous research conducted by our team found that both clinicians and users value a system that allows personalized interventions [35]. The CBT+ clinician portal offered multiple options to clinicians to personalize the content, timing, and frequency of interventions to their patients’ needs. For example, clinicians could craft personalized push notification interventions and used this feature in approximately 1 out of 5 sessions. These personalized push notifications were frequently used to target adherence to treatment recommendations (eg, reminder delivered every morning to remind participants to avoid at-home weighing) and to address treatment-interfering behaviors (eg, push notifications scheduled to remind participants every weekend to avoid high alcohol use to prevent the risk of binge eating).

Patients who completed treatment experienced improvements in CBT skills and significant reductions in binge eating and compensatory behaviors over the course of 16 weeks of treatment, and the average posttreatment EDE scores were in the remission range by the end of treatment. Although the additive efficacy of CBT+ cannot be directly assessed because of the lack of a control group, preliminary outcomes are promising and support the need for an RCT assessing whether CBT+ enhances outcomes when added to CBT for BN.

### Limitations and Future Work

Several limitations of this study must be acknowledged. First, we conducted an open trial, which limits our ability to draw conclusions about the effectiveness of CBT+ in improving skills acquisition and utilization above and beyond CBT alone. Second, our study had a small number of patients, as the primary goal of this pilot study was to evaluate the feasibility and acceptability of CBT+ ahead of an ongoing RCT. Our team is currently conducting a randomized control trial comparing CBT for BN with electronic self-monitoring via the CBT+ app without the JITAI system to CBT for BN with electronic self-monitoring via the CBT+ app with the JITAI system (ie, full CBT+ system) to better understand the additive value of CBT+. Third, although user adherence in our study was better than that observed in other mHealth app studies, we observed a modest decrease in user adherence in the latter half of treatment. Fourth, the dropout rate was relatively high. Although this rate is comparable with that reported in other treatment studies for individuals with BN (eg, 15%-65% across a range of other treatment studies [36-40]), these results suggest that there may be some participants for whom the CBT+ system is not feasible or acceptable. Fifth, as is typically the case in eating disorder studies, most participants were female; thus, app adherence and outcomes may not generalize to men. Sixth, similar to standard in-person CBT for BN [41], completing self-monitoring forms on the CBT+ app was perceived to be burdensome. Finally, although the interventions were generally found to be helpful, they were considered less useful later in treatment, likely because of their repetitive content.

Future iterations of the CBT+ app could better tailor JITAIs to improve their usefulness throughout the treatment. Similarly, the CBT+ app could incorporate and use more passive channels of data collection, such as continuous glucose monitoring, which could help increase the accuracy of the algorithm (eg, provide a risk alert to a participant when they have not eaten for more than 4 hours and are at a risk of binge eating) [42].

### Conclusions

In summary, our preliminary results suggest that the CBT+ system is a feasible and acceptable treatment augmentation for both patients and clinicians. If the results are confirmed in a larger RCT, the CBT+ system could prove to be a valuable addition to treatment to facilitate skill use and acquisition and ultimately reduce bulimic symptoms. Further research is necessary to determine the extent to which CBT+ is responsible for improving skills acquisition and utilization and whether improvements in skills acquisition and utilization mediate symptom reductions.

## Abbreviations

BN: bulimia nervosa
CBT: cognitive behavioral therapy
EDE: Eating Disorder Examination
JITAI: just-in-time adaptive intervention
NIMH: National Institute of Mental Health
RCT: randomized controlled trial
QOLI: Quality of Life Inventory

## References

[1] American Psychiatric Association (2013). Diagnostic and Statistical Manual of Mental Disorders. 5th Ed.

[2] Linardon J (2018). Meta-analysis of the effects of cognitive-behavioral therapy on the core eating disorder maintaining mechanisms: implications for mechanisms of therapeutic change. Cogn Behav Ther.

[3] Juarascio AS, Parker MN, Lagacey MA, Godfrey KM (2018). Just-in-time adaptive interventions: a novel approach for enhancing skill utilization and acquisition in cognitive behavioral therapy for eating disorders. Int J Eat Disord.

[4] Zendegui EA, West JA, Zandberg LJ (2014). Binge eating frequency and regular eating adherence: the role of eating pattern in cognitive behavioral guided self-help. Eat Behav.

[5] Barakat S, Maguire S, Surgenor L, Donnelly B, Miceska B, Fromholtz K, Russell J, Hay P, Touyz S (2017). The role of regular eating and self-monitoring in the treatment of bulimia nervosa: a pilot study of an online guided self-help CBT program. Behav Sci (Basel).

[6] Shah N, Passi V, Bryson S, Agras WS (2005). Patterns of eating and abstinence in women treated for bulimia nervosa. Int J Eat Disord.

[7] Ellison JM, Simonich HK, Wonderlich SA, Crosby RD, Cao L, Mitchell JE, Smith TL, Klein MH, Crow SJ, Peterson CB (2016). Meal patterning in the treatment of bulimia nervosa. Eat Behav.

[8] Wilson GT, Fairburn CC, Agras WS, Walsh BT, Kraemer H (2002). Cognitive-behavioral therapy for bulimia nervosa: time course and mechanisms of change. J Consult Clin Psychol.

[9] Towell D, Woodford S, Reid S, Rooney B, Towell A (2001). Compliance and outcome in treatment-resistant anorexia and bulimia: a retrospective study. Br J Clin Psychol.

[10] Thiels C, Schmidt U, Troop N, Treasure J, Garthe R (2001). Compliance with a self-care manual in guided self-change for bulimia nervosa. Eur Eat Disorders Rev.

[11] Troop N, Schmidt U, Tiller J, Todd G, Keilen M, Treasure J (1996). Compliance with a self-care manual for bulimia nervosa: predictors and outcome. Br J Clin Psychol.

[12] Lavender JM, Wonderlich SA, Engel SG, Gordon KH, Kaye WH, Mitchell JE (2015). Dimensions of emotion dysregulation in anorexia nervosa and bulimia nervosa: a conceptual review of the empirical literature. Clin Psychol Rev.

[13] Fischer S, Peterson CM, McCarthy D (2013). A prospective test of the influence of negative urgency and expectancies on binge eating and purging. Psychol Addict Behav.

[14] Peterson CB, Berg KC, Crosby RD, Lavender JM, Accurso EC, Ciao AC, Smith TL, Klein M, Mitchell JE, Crow SJ, Wonderlich SA (2017). The effects of psychotherapy treatment on outcome in bulimia nervosa: examining indirect effects through emotion regulation, self-directed behavior, and self-discrepancy within the mediation model. Int J Eat Disord.

[15] Nahum-Shani I, Smith SN, Spring BJ, Collins LM, Witkiewitz K, Tewari A, Murphy SA (2014). Just-in-time adaptive interventions (JITAIS): an organizing framework for ongoing health behavior support. University Park, PA: The Methodology Center, Penn State (Technical Report No. 14-126).

[16] Adams MA, Sallis JF, Norman GJ, Hovell MF, Hekler EB, Perata E (2013). An adaptive physical activity intervention for overweight adults: a randomized controlled trial. PLoS One.

[17] Thomas JG, Bond DS (2015). Behavioral response to a just-in-time adaptive intervention (JITAI) to reduce sedentary behavior in obese adults: Implications for JITAI optimization. Health Psychol.

[18] Kumar S, Abowd GD, Abraham WT, al'Absi M, Beck JG, Chau DH, Condie T, Conroy DE, Ertin E, Estrin D, Ganesan D, Lam C, Marlin B, Marsh CB, Murphy SA, Nahum-Shani I, Patrick K, Rehg JM, Sharmin M, Shetty V, Sim I, Spring B, Srivastava M, Wetter DW (2015). Center of excellence for mobile sensor data-to-knowledge (MD2K). J Am Med Inform Assoc.

[19] Haapala I, Barengo NC, Biggs S, Surakka L, Manninen P (2009). Weight loss by mobile phone: a 1-year effectiveness study. Public Health Nutr.

[20] Burke LE, Styn MA, Glanz K, Ewing LJ, Elci OU, Conroy MB, Sereika SM, Acharya SD, Music E, Keating AL, Sevick MA (2009). SMART trial: a randomized clinical trial of self-monitoring in behavioral weight management-design and baseline findings. Contemp Clin Trials.

[21] Beasley JM, Riley WT, Davis A, Singh J (2008). Evaluation of a PDA-based dietary assessment and intervention program: a randomized controlled trial. J Am Coll Nutr.

[22] Hamine S, Gerth-Guyette E, Faulx D, Green BB, Ginsburg AS (2015). Impact of mHealth chronic disease management on treatment adherence and patient outcomes: a systematic review. J Med Internet Res.

[23] Versluis A, Verkuil B, Spinhoven P, van der Ploeg MM, Brosschot JF (2016). Changing mental health and positive psychological well-being using ecological momentary interventions: a systematic review and meta-analysis. J Med Internet Res.

[24] Fairburn C (2008). Cognitive Behavior Therapy and Eating Disorders.

[25] Fairburn CG, Cooper Z, Shafran R (2003). Cognitive behaviour therapy for eating disorders: a "transdiagnostic" theory and treatment. Behav Res Ther.

[26] Cooper Z, Fairburn C (1987). The eating disorder examination: a semi-structured interview for the assessment of the specific psychopathology of eating disorders. Int J Eat Disord.

[27] Frisch MB (1994). Quality of Life Inventory® (QOLI®). National Computer Systems.

[28] Fairburn CG, Beglin SJ (1994). Assessment of eating disorders: interview or self-report questionnaire?. Int J Eat Disord.

[29] Gratz KL, Roemer L (2008). Multidimensional assessment of emotion regulation and dysregulation: development, factor structure, and initial validation of the difficulties in emotion regulation scale. J Psychopathol Behav Assess.

[30] Kordy H, Krämer B, Palmer RL, Papezova H, Pellet J, Richard M, Treasure J (2002). Remission, recovery, relapse, and recurrence in eating disorders: conceptualization and illustration of a validation strategy. J Clin Psychol.

[31] Le Grange D, Lock J, Agras WS, Moye A, Bryson SW, Jo B, Kraemer HC (2012). Moderators and mediators of remission in family-based treatment and adolescent focused therapy for anorexia nervosa. Behav Res Ther.

[32] Min YH, Lee JW, Shin Y, Jo M, Sohn G, Lee J, Lee G, Jung KH, Sung J, Ko BS, Yu J, Kim HJ, Son BH, Ahn SH (2014). Daily collection of self-reporting sleep disturbance data via a smartphone app in breast cancer patients receiving chemotherapy: a feasibility study. J Med Internet Res.

[33] Arean PA, Hallgren KA, Jordan JT, Gazzaley A, Atkins DC, Heagerty PJ, Anguera JA (2016). The use and effectiveness of mobile apps for depression: results from a fully remote clinical trial. J Med Internet Res.

[34] Torous J, Nicholas J, Larsen ME, Firth J, Christensen H (2018). Clinical review of user engagement with mental health smartphone apps: evidence, theory and improvements. Evid Based Ment Health.

[35] Juarascio AS, Goldstein SP, Manasse SM, Forman EM, Butryn ML (2015). Perceptions of the feasibility and acceptability of a smartphone application for the treatment of binge eating disorders: qualitative feedback from a user population and clinicians. Int J Med Inform.

[36] Agras W, Crow SJ, Halmi KA, Mitchell JE, Wilson GT, Kraemer HC (2000). Outcome predictors for the cognitive behavior treatment of bulimia nervosa: data from a multisite study. Am J Psychiatry.

[37] Fairburn CG, Jones R, Peveler R C, Hope R A, O'Connor M (1993). Psychotherapy and bulimia nervosa. Longer-term effects of interpersonal psychotherapy, behavior therapy, and cognitive behavior therapy. Arch Gen Psychiatry.

[38] Hoste RR, Zaitsoff S, Hewell K, le Grange D (2007). What can dropouts teach us about retention in eating disorder treatment studies?. Int J Eat Disord.

[39] Schnicker K, Hiller W, Legenbauer T (2013). Drop-out and treatment outcome of outpatient cognitive-behavioral therapy for anorexia nervosa and bulimia nervosa. Compr Psychiatry.

[40] Walsh BT, Wilson GT, Loeb KL, Devlin MJ, Pike KM, Roose SP, Fleiss J, Waternaux C (1997). Medication and psychotherapy in the treatment of bulimia nervosa. Am J Psychiatry.

[41] Wilson GT, Vitousek KM (1999). Self-monitoring in the assessment of eating disorders. Psychol Assess.

[42] Presseller EK, Parker MN, Lin M, Weimer J, Juarascio AS (2020). The application of continuous glucose monitoring technology to eating disorders research: an idea worth researching. Int J Eat Disord.

