# Authorship Correction: A Clinician-Controlled Just-in-time Adaptive Intervention System (CBT+) Designed to Promote Acquisition and Utilization of Cognitive Behavioral Therapy Skills in Bulimia Nervosa: Development and Preliminary Evaluation Study

**DOI:** 10.2196/31964

**Published:** 2021-07-29

**Authors:** Adrienne Juarascio, Paakhi Srivastava, Emily Presseller, Kelsey Clark, Stephanie Manasse, Evan Forman

**Affiliations:** 1 Center for Weight, Eating and Lifestyle Science Drexel University Philadelphia, PA United States

In “A Clinician-Controlled Just-in-time Adaptive Intervention System (CBT+) Designed to Promote Acquisition and Utilization of Cognitive Behavioral Therapy Skills in Bulimia Nervosa: Development and Preliminary Evaluation Study” (JMIR Form Res 2021;5(5):e18261), one error was noted.

In the originally published article, the authorship order was incorrect. The article was originally published with the following authorship order:

Adrienne Juarascio, Paakhi Srivastava, Kelsey Clark, Emily Presseller, Stephanie Manasse, Evan Forman

In the corrected article, the authorship order has been updated as follows:

Adrienne Juarascio, Paakhi Srivastava, Emily Presseller, Kelsey Clark, Stephanie Manasse, Evan Forman

The correction will appear in the online version of the paper on the JMIR Publications website on July 29, 2021, together with the publication of this correction notice. Because this was made after submission to PubMed, PubMed Central, and other full-text repositories, the corrected article has also been resubmitted to those repositories.

